# Precision in prostate cancer detection: integrating prostate-specific antigen density (PSAD) and the Prostate Imaging Reporting and Data System (PI-RADS) to provide additional risk stratification for a more accurate diagnostic decision

**DOI:** 10.1007/s11845-024-03771-w

**Published:** 2024-08-02

**Authors:** Terézia Hrubá, Viliam Kubas, Martin Franko, Vladimír Baláž, Martin Spurný, Jana Poláková Mištinová

**Affiliations:** 1 Radiology Department, F.D. Roosevelt University Hospital, Banská Bystrica, Slovakia; 2 Urology Clinic, Roosevelt University Hospital, Banská Bystrica, Slovakia; 3 M.R Institute, S.R.O, Banská Bystrica, Slovakia; 4 Radiology Clinic, Comenius University Bratislava, Bratislava, Slovakia

**Keywords:** MRI, PI-RADS, Prostate biopsy, Prostate cancer, PSA density

## Abstract

**Purpose:**

This study focuses on integrating prostate-specific antigen density (PSAD) and Prostate Imaging Reporting and Data System (PI-RADS) for enhanced risk stratification in biopsy-naïve patients.

**Methods:**

A prospective study was conducted on 339 patients with suspected prostate cancer, utilizing PSAD and PI-RADS in combination. Logistic regression models were employed, and receiver operating characteristic (ROC) analysis performed to evaluate predictive performance. The patient cohort underwent multiparametric MRI, targeted biopsy, and systematic biopsy.

**Results:**

When patients were stratified into four PSAD risk groups, the rate of clinically significant prostate cancer (csPCa) increased significantly with higher PSAD levels. Logistic regression confirmed the independent contribution of PI-RADS and PSAD, highlighting their role in the prediction of csPCa. Combined models showed superior performance, as evidenced by the area under the curve (AUC) for PI-RADS category and PSAD (0.756), which exceeded that of the individual predictors (PSA AUC, 0.627, PI-RADS AUC 0.689, PSAD AUC 0.708).

**Conclusion:**

This study concludes that combining PSAD and PI-RADS improves diagnostic accuracy and predictive value for csPCa in biopsy-naïve men, resulting in a promising strategy to provide additional risk stratification for more accurate diagnostic decision in biopsy-naïve patients, especially in the PI-RADS 3 group.

## Introduction

The detection of prostate cancer has long struggled with the challenges of overdiagnosis and subsequent overtreatment, forcing researchers to explore new approaches. The traditional diagnostic approach based on digital rectal examination (DRE), prostate-specific antigen (PSA) testing, transrectal ultrasound (TRUS). and systematic TRUS-guided biopsies often results in the identification of indolent tumors, leading to interventions of questionable clinical value.

In the quest for accuracy, magnetic resonance imaging (MRI) has emerged as a transformative tool in prostate cancer diagnosis. Unlike conventional imaging modalities, MRI provides detailed visualization of the prostate, enabling radiologists to more accurately identify suspicious lesions, grade them according to the Prostate Imaging Reporting and Data System (PI-RADS) v2.1 protocol, assigns lesions to grade groups ranging from 1 to 5, with higher grades indicating a higher likelihood of clinically significant cancer, and precisely localize them within the prostate. The integration of magnetic resonance imaging (MRI) and transrectal ultrasound (TRUS) images paves the way for targeted biopsy, combining the strengths of both modalities. This precise and focused approach is designed to sample areas of concern identified by MRI, reducing the risk of overdiagnosis by targeting clinically significant tumors.

The proposal to use prostate MRI as a stratification method before biopsy requires the demonstration of a robust negative predictive value. This is essential to provide confidence that potentially lethal cancers will not go undetected. However, the reported negative predictive value of MRI for prostate cancer has shown considerable variability between studies, ranging from 63 to 100%, which has been attributed to differences in cancer prevalence between the cohorts studied [8]. It is also important to acknowledge the existing interobserver variability in MRI reporting between radiologists using the PI-RADS protocol, which may limit the value of MRI in determining which patients should undergo prostate biopsy [7, 12]. Furthermore, in the field of prostate cancer management, the classification of lesions as PI-RADS category 3 introduces an area of uncertainty where the balance between overtesting and detection of csPCa needs to be carefully considered [5, 6, 11].

Recognizing this challenge, the goal of this study is to explore if the combination of the PI-RADS protocol and prostate-specific antigen density (PSAD) can differentiate between indolent and clinically significant prostate cancer in biopsy-naïve patients and provide additional risk stratification for a more accurate diagnostic decision. To address this query, we used methodology of existing study [12].

## Patients and methods

We conducted a prospective study approved by the local Institutional Ethical committee, number 28/2021.

### Patient population

We analyzed the data of 538 patients with suspected prostate cancer with PI-RADS-3 score or higher from January 2021 to May 2023 at the Urology Clinic of F.D. Roosevelt University Hospital, Banská Bystrica, Slovakia. We included patients who had undergone MRI with findings of PI-RADS 3 lesion or higher. Subsequently, all patients underwent MRI/TRUS fusion targeted biopsy followed by standard systematic biopsy with 10 samples. Patients who were lacking data about serum prostate-specific antigen (PSA) level, prostate glandular volume measurements, or histology results from biopsies recorded in the registry were excluded. Also, patients who underwent rebiopsy or were in active surveillance were excluded. The final study consisted of 339 biopsy-naïve patients.

### Prostate MRI technique and interpretation

All patients underwent multiparametric magnetic resonance imaging (mpMRI) before the biopsy procedure, conducted in multiple magnetic resonance departments on either a 1.5 T (Magnetom Essenza, Siemens Healthineers) or 3 T (Magnetom Spectra, Magnetom Verio, Siemens Healthineers) machine, utilizing a 24-channel pelvic phased array coil. The image acquisition protocol adhered to the guidelines outlined in PI-RADS v2.1. Glandular volume was determined using a 3D T2-weighted pulse sequence; the PSA density (PSAD) was calculated by dividing the serum PSA by the prostate gland volume employing a manual or semi-automated procedure through syngo.via.

Evaluation of the mpMRIs was performed by one of 20 radiologists, each possessing a minimum of 5 years of experience in abdominal MRI, which includes expertise in prostate imaging. The assessment utilized the PI-RADS scoring system v2.1 to characterize and describe the findings obtained from the magnetic resonance imaging scans.

For the purpose of the data analysis, the study population was stratified into four PSAD risk groups: < 0.10, 0.10–0.15, 0.15–0.20, and > 0.20 ng/mL/mL [12].

### Prostate biopsy and pathology analysis

The MRI-targeted prostate biopsies were conducted by two urologists (V.K. and M.F.) using Hitachi Arietta v70® with MRI and transrectal ultrasound rigid fusion software. Patients with at least one lesion having a PI-RADS score of 3 or higher underwent both MRI-targeted biopsy and a 10-core systematic biopsy. A radiologist (T.H.) marked the prostate and suspect lesions in the ultrasound software based on prior descriptions by radiologists using T2-weighted and diffusion-weighted imaging (DWI) MRI sequences.

Transrectal prostate biopsies were performed using an automated biopsy gun (BARD Magnum) and an 18-G needle. The number of targeted lesions varied from 1 to 2, with two cores obtained per target. Uropathologists reviewed the biopsy samples, and prostate cancer findings were graded according to the Gleason score and the corresponding International Society of Urological Pathology (ISUP).

For the scope of this study, the ISUP grade groups from both MRI-targeted biopsy and a 10-core systematic biopsy were evaluated and the highest ISUP grade was attributed from combination of both types of biopsies. Prostate cancer with a grade group of ISUP 2 or higher was categorized as clinically significant prostate cancer (csPCa). Clinically insignificant or indolent prostate cancer, defined by ISUP as Grade 1, is equivalent to a Gleason score of 6 or less. It is a low-risk, slow-growing cancer that usually does not spread or cause significant health problems, and recommended management options are active surveillance (AS) or watchful waiting (WW) [4].

The classification of mpMRI results was determined based on the lesion with the most severe PI-RADS score.

### Statistical analysis

The factors that were assessed for the risk of a positive biopsy included the following: PSA level, prostate volume, PSA density, PI-RADS v2.1 score, and TRUS biopsy findings, using Excel tables and figures.

Logistic regression models were fitted for each of PI-RADS category, PSA, and PSAD as independent variables and with binary csPCa as dependent variable. In addition, two models were fitted with two explanatory variables: (1) PI-RADS category and PSA and (2) PI-RADS category and PSAD.

ROC curves were constructed using predicted probabilities from the five logistic regression models. The area under the curve (AUC) was calculated for each model. The models with three higher AUC were compared using nonparametric methods [3]. All analyses were performed in R version 4.3.0 [9].

## Results

### Patient and clinical characteristics

The study comprised a diverse group of 339 biopsy-naive patients. The age of this group was ranging from 47 to 84 years.

The mean PSA level for the entire cohort is 9.23 ng/mL. The mean MRI prostate volume is 58.7 mL, ranging from 15 to 221 mL. The mean PSAD for the entire study is 0.18 ng/mL^2^, with a range from 0.02 to 2.03 ng/mL^2^.

Analysis of the PI-RADS distribution shows that PIRADS 4 is the most common category with 51% in biopsy-naïve patients (Table 1).

**Table 1:** Patient and clinical characteristics

Characteristic	Values
Study patient population	339
Year 2021	110
Year 2022	157
Year 2023	72
Age mean (range)	65.7 (47–84)
Age < 50	7
Age 50–60	64
Age 60–70	161
Age 70–80	100
Age > 80	7
PSA mean (range) ng/mL	9.23 (0.9–110)
Volume mean (range) mL	58.7 (15–221)
PSAD mean (range) ng/mL^2^	0.18 (0.02–2.03)
PI-RADS *n* (%)	
3	91 (27%)
4	174 (51%)
5	74 (22%)
NoCarc	114
ISUP 1	68
ISUP 2	99
ISUP 3	29
ISUP 4	25
ISUP 5	4

**Fig. 1:**
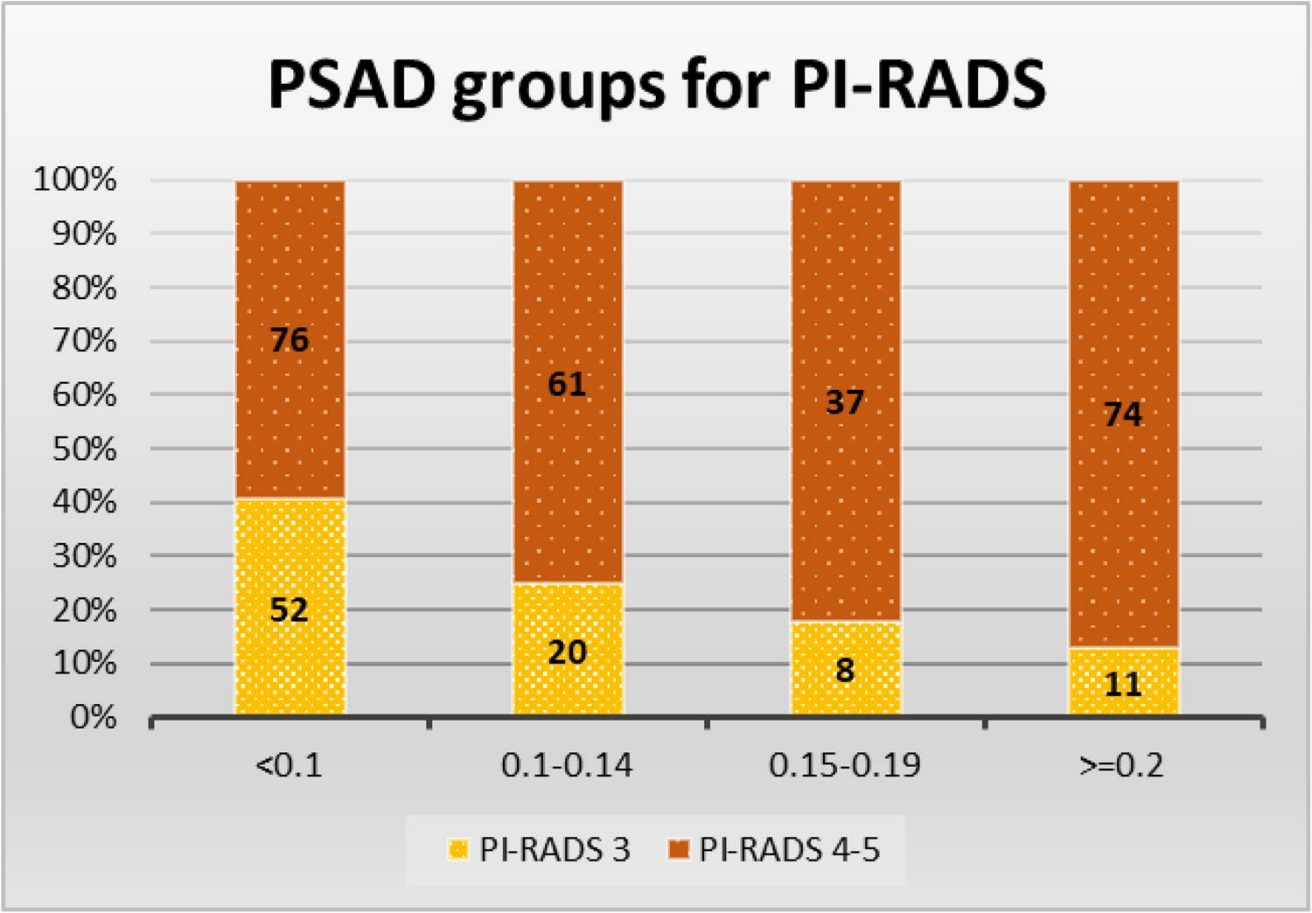
Distribution of MRI results according to PI-RADS score for four risk groups of PSAD, (*N* = 339), years 2021–2023

For the study population of patients, in the first step, we stratified MRI results according to Prostate Imaging Reporting and Data System (PI-RADS) scores into four risk groups of prostate-specific antigen density (PSAD) and in the second step, we stratified biopsy results (ISUP) into four risk groups of prostate-specific antigen density (PSAD) and Prostate Imaging Reporting and Data System (PI-RADS) scores and calculated the percentages and counts for each stratum, providing insight into the relationship between imaging findings and PSAD levels [10] (Table 2, Fig. 1, Table 5).

**Table 2:** Stratification of MRI results according to PI-RADS score for four risk groups of PSAD (*N* = 339), years 2021–2023

PI-RADS	PSAD for PI-RADS							
	Low risk < 0.10		Low–intermediate risk 0.10–0.14		Intermediate–high risk 0.5–0.19		High risk 0.20 and more	
	%	*N*	%	*N*	%	*N*	%	*N*
PI-RADS 3	40.6%	52	24.7%	20	17.8%	8	12.9%	11
PI-RADS 4–5	59.4%	76	75.3%	61	82.2%	37	87.1%	74

**Fig. 2:**
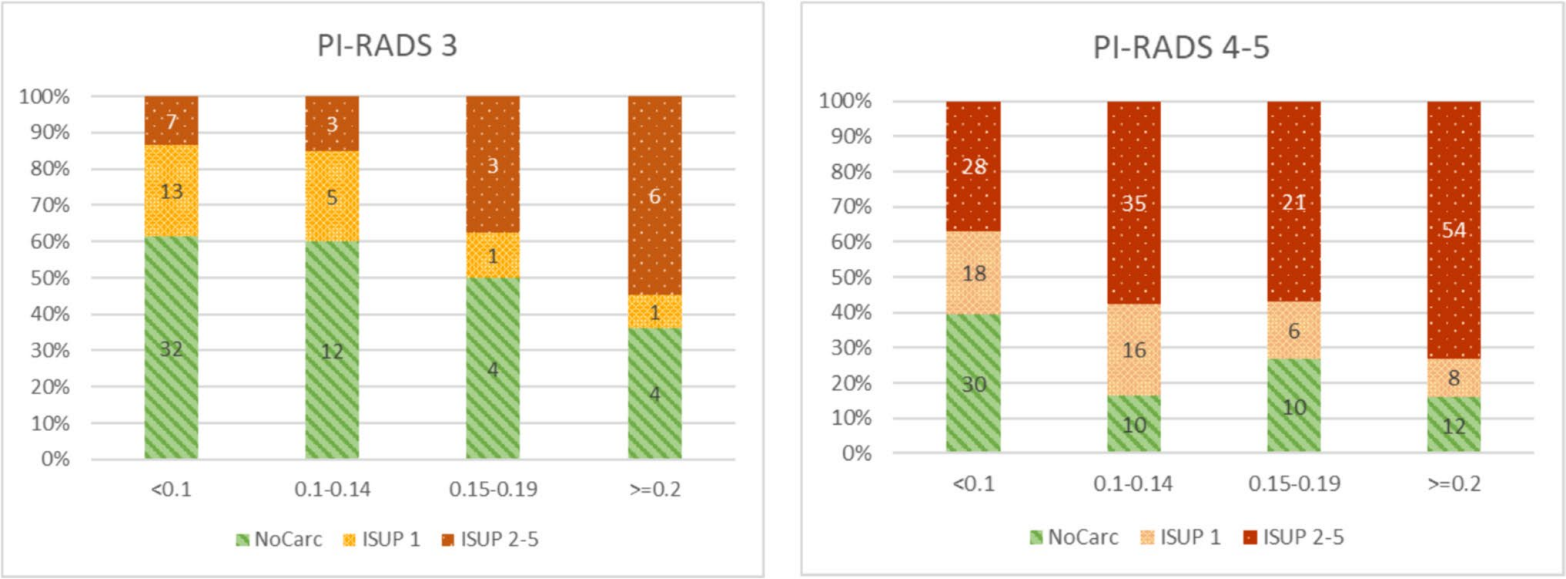
Distribution of biopsy results by PSAD group levels according to MRI results in diagnosis. **A** PI-RADS 3 group of patients (*N* = 91). **B** PI-RADS 4–5 group of patients, (*N* = 248), years 2021–2023

Men in the PI-RADS 3 MRI group were overall less likely to have diagnosed csPCa on biopsy—20.9%. When analyzing the csPCa by PSAD strata, men is this MRI group and low-risk PSAD < 0.10 were the least likely group to have positive biopsy—13.5%. This proportion increased to 15% in the stratum of low-intermediate-risk PSAD 0.10–0.14, further to 37.5% in stratum with intermediate-high risk PSAD between 0.15 and 0.19, and increased to 54.5% in stratum with high risk PSAD 0.2 and higher (Table 3, Fig. 2A).

**Table 3:** Stratification of biopsy results (ISUP) by four risk groups of PSAD in PI-RADS 3 group of patients (*N* = 91), years 2021–2023

ISUP groups	PSAD for PI-RADS 3 group *N* 91							
	Low risk < 0.10		Low–intermediate risk 0.10–0.14		Intermediate–high risk 0.15–0.19		High risk > 0.20	
	%	*n*	%	*n*	%	*n*	%	*n*
NoCarc	61.5%	32	60.0%	12	50.0%	4	36.4%	4
ISUP1	25.0%	13	25.0%	5	12.5%	1	9.1%	1
ISUP2-5	13.5%	7	15.0%	3	37.5%	3	54.5%	6

Men in the PI-RADS 4–5 MRI group were more likely to have diagnosed csPCa on biopsy—55.6%. By PSAD strata, csPCa for low-risk PSAD < 0.1 was diagnosed in 36.8%, for low-intermediate-risk PSAD between 0.10 and 0.15 in 57.4%, and for intermediate-high-risk PSAD between 0.15 and 0.19 in 56.8%, and the highest proportion of 73.0% was for stratum with high-risk PSAD 0.2 and higher (Table 4, Fig. 2B).

**Table 4:** Stratification of biopsy results (ISUP) by four risk groups of PSAD in PI-RADS 4 group of patients (*N* = 248), years 2021–2023

ISUP groups	PSAD for PI-RADS 4–5 group *N* 248							
	Low risk < 0.10		Low–intermediate risk 0.10–0.14		Intermediate–high risk 0.15–0.19		High risk > 0.20	
	%	*n*	%	*n*	%	*n*	%	*n*
NoCarc	39.5%	30	16.4%	10	27.8%	10	16.2%	12
ISUP1	23.7%	18	26.2%	16	16.7%	6	10.8%	8
ISUP2-5	36.8%	28	57.4%	35	56.8%	21	73.0%	54

PI-RADS 3 MRI group and PI-RADS 4–5 MRI groups of patients demonstrate an increasing prevalence of csPCa as PSAD levels rise (Fig. 1) (Table 5). These results were confirmed by logistic regression, where odds of csPCa detection was associated with increases of PI-RADS category (*p* < 0.001), PSAD (*p* < 0.001), and also PSA (*p* = 0.007) (Table 6).

**Table 5:** Proportion of clinically significant prostate cancer (ISUP2-5) detected by biopsy according to MRI results divided in four risk group of PSAD (*n* = number of ISUP2-5, *N* = number in PI-RADS groups, patients 339), years 2021–2023

PI-RADS groups	Clinically significant prostate cancer									
	Total counts		Prostate-specific antigen density (PSAD)							
			Low risk < 0.10		Low–intermediate risk 0.10–0.14		Intermediate–high risk 0.15–0.19		High risk > 0.20	
	%	(*n*/*N*)	%	(*n*/*N*)	%	(*n*/*N*)	%	(*n*/*N*)	%	(*n*/*N*)
PI-RADS 3 group	20.9%	19/91	13.5%	7/52	15.0%	3/20	37.5%	3/8	54.5%	6/11
PI-RADS 4–5 group	55.6%	138/248	36.8%	28/76	57.4%	35/61	56.8%	21/37	73.0%	54/74
All PI-RADS	46.3%	156/339	26.3%	35/128	46.9%	38/81	53.3%	24/45	70.6%	60/85

**Table 6:** Odds ratio for clinically significant prostate cancer for biopsy-naive group of patients (*N* = 339), years 2021–2023

Predictor included in the model	OR^1^	95% CI^1^	*p*-value
PI-RADS category (increase of 1)	3.08	2.18, 4.45	< 0.001
PSAD (increase of 0.05)	1.35	1.21, 1.53	< 0.001
PSA (increase of 1)	1.04	1.01, 1.08	0.007

^1^*OR* odds ratio, *CI* confidence interval

The AUC for two predictors PI-RADS category and PSAD was the highest (0.756), with small significant increase when compared to the AUC for PSAD (0.708) (Table 7). The difference was 0.049 with 95% confidence interval (CI) 0.006, 0.091, and *p*-value 0.026. Similarly, the AUC for two predictors PI-RADS category and PSAD was significantly higher compared to AUC of PI-RADS category with PSA (difference:0.036; 95% CI 0.012, 0.06; *p*-value 0.003) (Table 7).

**Table 7:** AUC for clinically significant prostate cancer detection, biopsy-naive group of patients (*N* = 339), years 2021–2023

Predictor(s) included in model	AUC^1^	95% CI^1^
PSA	0.627	0.567, 0.686
PI-RADS category	0.689	0.638, 0.739
PSAD	0.708	0.653, 0.762
PI-RADS category + PSA	0.720	0.666, 0.774
PI-RADS category + PSAD	0.756	0.706, 0.807

^1^*AUC* area under the curve, *CI* confidence interval

## Discussion

The comprehensive analysis of 339 men found that the combination of prostate-specific antigen density (PSAD) and the Prostate Imaging Reporting and Data System (PI-RADS) improves the performance of the PI-RADS protocol in prostate cancer diagnosis in biopsy-naïve patients and can help in the risk decision-making process before prostate biopsy.

Following the methodology of existing study, the population was stratified into four PSAD risk groups: < 0.10, 0.10–0.15, 0.15–0.20, and > 0.20 ng/mL/mL, with csPCa detection rates of 26.3%, 46.9%, 53.3%, and 70.6% of the total population, respectively [12].

In the PI-RADS 3 MRI group, where diagnostic uncertainty is common, the likelihood of clinically significant prostate cancer (csPCa) exhibited a notable escalation with higher PSAD levels. The percentage of csPCa in this group increased from 13.5% for low-risk PSAD < 0.10 to 54.5% for high-risk PSAD 0.2 and higher, demonstrating a clear association between elevated PSAD and an increased risk of significant malignancy.

In the low-risk PSAD group (< 0.10 ng/mL/mL) consisting of 52 men, seven men had diagnosed csPCa from biopsy, of which six were ISUP 2 and one ISUP 3, putting them in intermediate–low-risk group with 13.5% chance of presence of csPCa, these men may avoid immediate biopsy only with an adequate monitoring as part of shared decision-making [4, 13]. The high-risk PSAD group (> 0.20 ng/mL/mL) have an increased risk of csPCa of 54.5%, so a biopsy should be performed.

Within the MRI group classified as PI-RADS 4–5, the occurrence of clinically significant prostate cancer (csPCa) consistently showed higher rates across all prostate-specific antigen density (PSAD) categories. The highest proportion, reaching 73.0%, was observed in the high-risk PSAD (> 0.20 ng/mL/mL) subgroup. According to European Association of Urology (EAU) recommendations, individuals falling into this category should undergo targeted biopsy, either alone or in combination with systematic biopsy. Conversely, in the low-risk PSAD group (< 0.10 ng/mL/mL), 36.8% of individuals were diagnosed with csPCa, indicating that biopsies should be performed in men with low PSAD levels if MRI scans are positive.

When comparing our results with those of a similar study with a cohort of 3006 patients, men with PI-RADS 3 scores and low-risk PSAD (< 0.10 ng/mL/mL) had a lower risk (4%) of significant disease than in our study, suggesting that biopsies could have been avoided altogether, because our risk was higher—13%—we suggest avoiding biopsy only under close monitoring. The high-risk PSAD group (> 0.20 ng/mL/mL) had an increased risk of csPCa of 29%; in our study, the risk was even higher at 54%, so a biopsy should be done anyway [11].

According to various studies, PSAD does not influence biopsy decision in PI-RADS 4–5 category due to the higher prevalence of csPCa in these patients, so it was not found to be a useful tool to prevent biopsy in these patients; our results are consistent with these studies [11, 14].

**Fig. 3:**
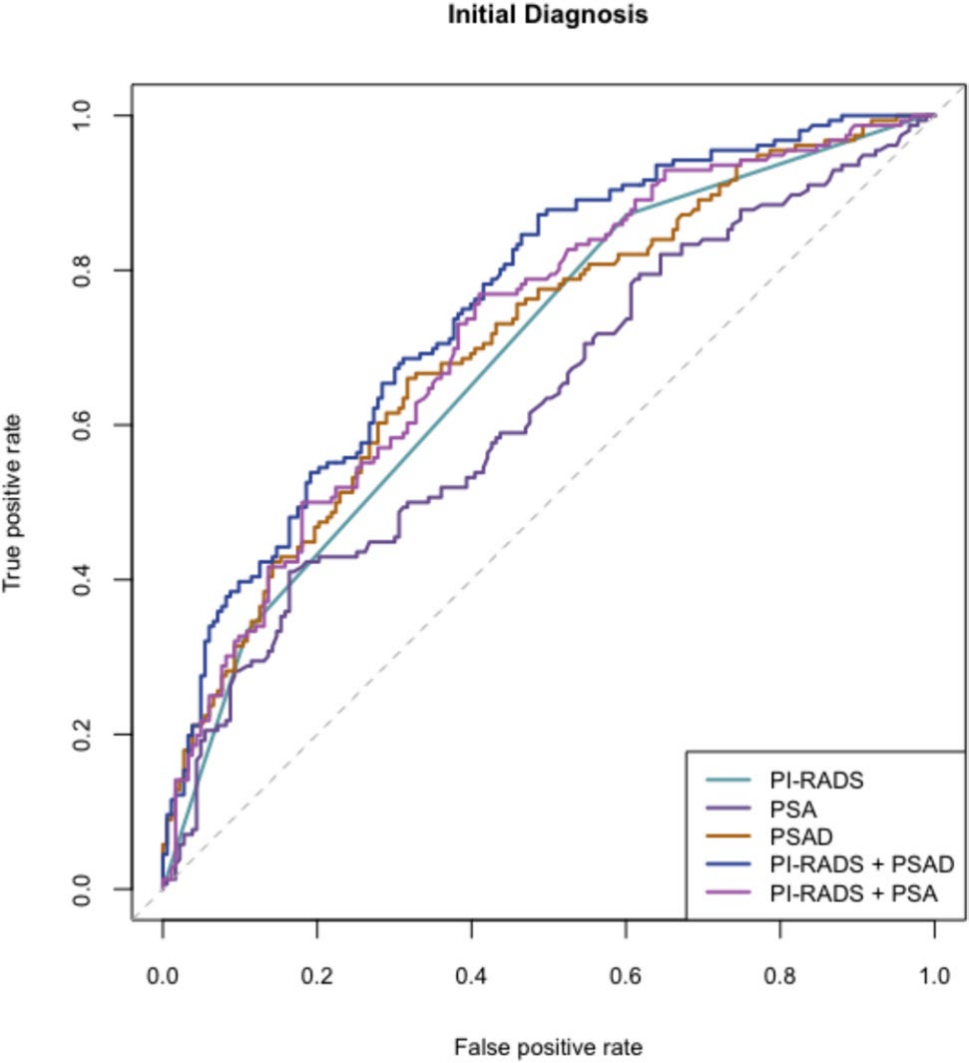
ROC curves for the biopsy-naive group of patients (*N* = 339) based on csPCa cases detected by combined biopsy from logistic regression models, years 2021–2023. Predictors are listed in the legend

Logistic regression affirmed the independent contributions of both PI-RADS category and PSAD levels to the likelihood of detecting csPCa. This statistical confirmation underscores the importance of integrating advanced imaging and biomarker data for a comprehensive risk assessment. The ROC analysis further emphasized the superior predictive performance of the combined model, where the AUC for PI-RADS category and PSAD (0.756) surpassed the individual predictors (PSA AUC 0.627, PI-RADS AUC 0.689, PSAD AUC 0.708.) The statistical significance of the differences in AUC values adds robustness to the argument for the synergistic value of PSAD and PI-RADS in predicting csPCa (Fig. 3). According to the results, the optimal PSAD cut-off of 0.10 ng/mL/mL is recommended in men with equivocal MRI results (PI-RADS 3) to determine the need for biopsy.

Our findings are consistent with the results of several other studies that investigated the predictive value of PSAD in conjunction with MRI outcomes [1, 12, 15]. In the biopsy-naïve subset of the entire cohort comprising 526 patients, the incorporation of PI-RADS with PSAD resulted in an increased area under the curve (AUC) (PI-RADS score AUC = 0.799 vs. PI-RADS score + PSAD AUC = 0.830, difference in AUC = 0.031, 95% CI 0.012, 0.050, *p* = 0.002). However, such an effect was not observed in the active surveillance group (PI-RADS score AUC = 0.762 vs. PI-RADS score + PSAD AUC = 0.778, difference in AUC = 0.016, 95% CI 0.040, 0.071, *p* = 0.579) [12]. In a cohort of 372 men of comparable size, logistic regression models were formulated. The combination of the PI-RADS score and PSAD (AUC = 0.80) demonstrated a significantly superior performance compared to both PSA (AUC = 0.75, *p* < 0.01) and the Prostate Cancer Prevention Trial (PCPT) risk calculator (AUC = 0.76, *p* < 0.01). This combination substantially enhanced the risk discrimination for clinically significant prostate cancer on biopsy [1]. Within a cohort of 833 biopsy-naïve men, the optimal diagnostic performance for detecting csPCa was achieved through the combination of the PI-RADS score and PSAD. The AUC for the combined PI-RADS score with PSAD was 0.942 (95% CI 0.924–0.957). Notably, the diagnostic accuracy surpassed that of any individual clinical variable, including the PI-RADS score (*p* < 0.001) [15].

The study presents certain limitations that should be acknowledged. Despite efforts to involve experienced radiologists, the interobserver variability in MRI reporting may impact result consistency. The classification of lesions as PI-RADS category 3 introduces uncertainty in decision-making, requiring careful consideration of the balance between overtesting and detecting clinically significant cancer. Exclusion criteria, including patients under active surveillance and those with missing essential data, may introduce selection bias. The study’s single-center nature limits generalizability, and the relatively short follow-up until May 2023 restricts the assessment of long-term outcomes. Additionally, the absence of external validation in diverse cohorts and geographic regions emphasizes the need for cautious interpretation and potential adjustments when applying the combined PSAD and PI-RADS diagnostic approach.

Furthermore, an acknowledged challenge within the field is the interobserver variability in MRI reporting, a factor that could potentially introduce biases. The reliance on subjective interpretations, even by experienced radiologists, underscores the need for ongoing efforts to standardize reporting practices and mitigate potential variations in assessments. As the field advances, collaborative initiatives and standardized reporting protocols will be essential to enhance the robustness and reliability of future studies in prostate imaging.

## Conclusion

The integration of PSAD and PI-RADS improves the diagnostic accuracy and predictive value for csPCa in biopsy-naïve men, resulting in a promising strategy to provide additional risk stratification for a more accurate diagnostic decision in biopsy-naïve patients, especially in the PI-RADS 3 group. In men with equivocal MRI results (PI-RADS 3), using a PSAD cut-off of 0.10 ng/mL/mL to recommend biopsy appeared to be the best strategy; men with PSAD levels < 0.10 ng/mL/mL can avoid biopsy with appropriate surveillance, based on the results from our patient cohort. Larger studies are needed to validate our findings in other patient populations.
